# The Finis of “War Medicine”

**Published:** 1919

**Authors:** 

**Affiliations:** War Medicine, and Secretary, Research Committee of the American Red Cross in France


### WAR MEDICINE

Published by the American Red Cross Society in France
for the
Medical Officers of the American Expeditionary Forces

**EDITORIAL OFFICES :** **2, Place de Rivoli, Paris, Room 425.**

WAR MEDICINE *accepts no original articles. Its scope is limited to the publication of the proceedings of the Research Society of the American Red Cross in France, abstracts of original articles, and editorial comment on subjects pertaining to the medicine, surgery, and hygiene of the war.*
*Circulars, bulletins, and reports from the Office of the Chief Surgeon of the American Expeditionary Forces will also appear in* WAR MEDICINE.

WAR MEDICINE *is distributed free of charge to the medical officers of the American Expeditionary Forces.*
*All communications pertaining to the mailing list should be addressed to the Editorial Offices. If copies are not received promptly and regularly the managing editor should be informed at once. Addresses should be written distinctly.*

THE RESEARCH SOCIETY OF THE AMERICAN RED CROSS IN FRANCE
*All communications should be addressed to the Secretary,*
*2, Place de Rivoli, Paris, Room 425*

### EDITORIAL COMMENTS

### THE FINIS OF " WAR MEDICINE ".

The monthly medical journal, formerly called the *Medical Bulletin*, and now known as *War Medicine*, which has been published by the American Red Cross for the medical officers of the American Expeditionary Forces, will be discontinued with this, the February-March number. The object of this journal has been the dissemination of some of the latest and most important information on subjects pertaining to medicine, surgery and hygiene, as applied to war conditions. If it has been in any way helpful to those who have been privileged to treat the wounds and to care for the health of our American soldiers, and those of our Allies, then the aims and purposes of those responsible for, and interested in, the publication of an American medical journal in France for the period of the war have been attained.

Excepting the department of abstracts, perhaps the greatest value of *War Medicine* comes from the fact that it has been the medium for publishing the transactions of the Research Society of the American Red Cross in France, which brought together each month for more than a year many of the leading authorities on subjects pertaining to the various phases of war medicine. Our medical confreres in the French, British and Italian Armies have been good enough to lay before the Research Society their views on the many problems with which they have had to deal during the four tragic years that they have been at war, and which were new to the medical officers of the American Expeditionary Forces. Furthermore, many American medical officers have outlined at these meetings plans and methods of handling the various medical problems of the American Expeditionary Forces. The two hundred or more medical officers who attended these meetings each month derived the greatest benefit from them, but the thousands of others who have read in *War Medicine* the reports of the Research Society meetings no doubt have acquired much useful knowledge from the experience of those who have had such vast opportunities of dealing with the medical problems of the war.

### Hostilities Continue in the Medical Depatment.

When hostilities ceased, many medical officers returned to the United States, and this fact, coupled with the unsettled conditions in the America Expeditionary Forces generally, caused the discontinuance of the meetings of the Research Society to be deemed advisable; and since *War Medicine* was being published for the purpose of disseminating medical information pertaining to the war, it was felt that with the cessation of hostilities the sphere of action of this journal was considerably diminished, except perhaps as regards the summing up of some of the experiences of the medical officers of the American Expeditionary Forces for historical purposes.

It was therefore decided that the publication of *War Medicine* should cease with the February-March number. This does not mean that those responsible for *War Medicine* feel that the work of the medical officers in France is ended, and that medical journals are not needed as much now as during the war; on the contrary, we know that General Streptococcus and his Armies, and that other myriad hosts of disease-producing germs have not ceased hostilities. The Medical Department of the American Expeditionary Forces will have to keep up the fight against preventable diseases until the last American soldier has embarked for the United States.

The medical problems of an army in times of peace are much the same as those of a community in civil life where large numbers of men are assembled together. There are many excellent medical journals which cover this field, and therefore the Medical Department of the Army and of the American Red Cross have subscribed for a number of medical periodicals which will be sent to various hospitals where they will be of value to the medical officers connected with them. In this way the need for medical journals (" text-books ") will be supplied, and since physicians in the Army may now be expected to have more leisure, it is hoped that they will read more medicine than ever before.

### A Word of Appreciation.

After an author has completed a book, or an editor has compiled one, it is customary to write a preface, or an introduction, expressing appreciation to those who have been associated with him, and giving credit to those who deserve it for their part in producing the publication. It would therefore seem not inappropriate, as this number marks the end of the publication known as *War Medicine*, to mention briefly the names and the merit of those who deserve praise for their work in connection with the *Medical Bulletin* and *War Medicine*.

The wisdom and assistance of Colonel Alexander Lambert, Chief of the Department of Medical Research and Intelligence of the American Red Cross in France, was indispensable to the success of the *Medical Bulletin* and *War Medicine*.

He had the vision to see the need of such a publication, and to provide, through the Red Cross, the ways and means for the building-up of the organization necessary to carry on the publication of a medical journal in France. Lieutenant-Colonel Strong, who succeeded Colonel Lambert when the latter returned to the United States in December, has been equally interested in the medical publications of the American Red Cross.

The members of the Research Committee have been the inspiration for *War Medicine.* By their encouragement and their assistance, when needed, the work has been carried on successfully, we hope. Some of the members of the Research Committee, in addition to compiling the reports of the various conferences of the Research Society, have contributed editorials that have added to the tone and the value of the publications intended for the American physicians and surgeons serving in France.

Surgeon-General Ireland, who was a member of the Research Committee, showed great personal interest in the up-building of *War Medicine*, and it was by his authority that it became an official publication for the medical officers of the American Expeditionary Forces. Chief-Surgeon McCaw has continued the policy of his predecessor. Lieutenant-Colonel Haven Emerson of the Chief Surgeon's office has also rendered valuable assistance to the Editor.

### The Medical Bulletin.

Those who were with the American Expeditionary Forces in the early months of its activities in France will recall that the first number of the *Medical Bulletin* was published in November 1917. Major Kenneth Taylor, in charge of the laboratory of American Military Hospital No. 2, which had working in conjunction with it a Research Laboratory of the American Red Cross, although weighted with work, gave the time necessary for the editing of this monthly publication, the *Medical Bulletin*, until May 1918. By this time, Major Taylor's duties connected with his laboratory and research work had so increased that he was unable to continue to give the time necessary for editorial work, and he requested that a medical officer be detailed to devote all of his time and energies to the double work of Editor of *War Medicine* and Secretary of the Research Committee. Then it was that I had the good fortune to be called to France to undertake this work.

The *Medical Bulletin*, under the guidance of Major Taylor and Mr. E. Bradlee Watson (Ph. D. Harvard), Managing Editor, had been such a success and of so much value to the medical officers of the American Expeditionary Forces, that the continuance of the journal was not difficult. Of course there have been difficulties in getting together an adequate staff to carry on a monthly medical journal of 150-250 pages, but there have been many fine men and women who have sacrificed to serve with the American Red Cross, and I have not had the good fortune hitherto to serve with a more delightful, harmonious and hard-working group of men and women than those with whom I have been associated in the Bureau of Medical Publications of the American Red Cross in France.

### War Medicine.

Too much credit cannot be accorded to Mr. E. Bradlee Watson, who, prior to America's entry into the war, was Dean and Professor of English at Robert College, Constantinople, for his work as Managing Editor of *War Medicine*. He had charge of the business side of the Bureau of Medical Publications, and although not a physician he read the proofs and assisted in editing the abstracts for the *Medical Bulletin* and *War Medicine*, and in many other ways was indispensable in the publication of a magazine in a foreign land. Dr. Watson has now returned to take up his duties at Robert College.

Sergeant Jacques Magnin of the Medical Corps of the American Expeditionary Forces, who was a medical student in Paris at the outbreak of the war, was connected with the *Medical Bulletin* from its beginning, and did excellent work in abstracting. He has now returned to his medical studies in Paris. My Secretary, Miss Theodora Stonehouse, an English woman, who has a thorough knowledge of French, has aided greatly in the work of *War Medicine*, as have Misses Elsie and Olive Tarbell and Miss Helen Brown, who came from Darmouth College, and Miss Clendennen, of Richmond, Va., who have all worked with the Bureau of Medical Publications. Madame Rivière, Mesdemoiselles Boussard and Sailly, have also given valuable assistance in abstracting foreign medical journals. Miss Jameson of the New York Public Library, who has been with the Bureau of Medical Intelligence of the American Red Cross, has also aided greatly by her intelligent efforts in looking up references in medical literature. The publishers of *War Medicine,* Messrs. Masson et Cie, the largest medical publishing house in France, have given us most cordial co-operation. Even when paper was almost unobtainable, Masson et Cie succeeded in getting enough together to publish *War Medicine* and other medical publications for the Red Cross, although in so doing they had to curtail their other publications.

### Difficulties and Delays.

There have been many difficulties and regrettable delays in publishing *War Medicine,* which would not have occurred in normal times. At the time when they occurred, they appeared to be serious obstacles, but now that it is all over, they seem to have been publication troubles that were not so important after all. The greatest difficulty has been in the distribution of *War Medicine*, but when one considers the transportation problems of moving millions of troops and the necessary food supplies and munitions of war for them, it is wonderful that non-essentials — as some consider medical journals — were transported at all.

The effort has been made for the past five months to supply each medical officer in the American Expeditionary Forces with a personal copy of *War Medicine*, but because of the many changes in the location of various army units, a considerable number of the copies which had been sent to addresses provided by the Chief Surgeon's Office have not been delivered, and many of these have been returned to the Bureau of Medical Publications of the Red Cross.

We have on hand a number of copies of very nearly complete files of the *Medical Bulletin* and *War Medicine.* Most of them will be kept at the American Red Cross Medical Library, 12, Place Vendome, Paris, but some will be sent to the American Red Cross at Washington for distribution among medical officers who have returned to the States. Letters applying for back numbers of *War Medicine* should be addressed to the American Red Cross Medical Library, 12, Place Vendome, Paris, or to me c/o The Office of the Surgeon General, Washington, D. C., where I shall be until I have completed the chapters on digestive diseases in the Medical History of the War, which is now being prepared in in Washington.

It has been a glorious privilege to serve in France during the greatest crisis in the world's history, and if I have had a part in carrying to the medical officers who have served at the front, or anywhere else with the American Expeditionary Forces, an idea that has aided them in their work, I feel that I have not served in vain.

Of course I am glad that the war is over, and that there is no longer a need for *War Medicine*, so that I can return to my family and to the practice of my profession in civil life; but it is with genuine regret that I am leaving the medical officers with whom and for whom I have served during the eight months of the most wonderful year that has passed.

SEALE HARRIS, Lt.-Col., M. C.,
Editor, *War Medicine*, and Secretary, Research Committee of the American Red Cross in France.

